# Where Is Current Research on Blockchain Technology?—A Systematic Review

**DOI:** 10.1371/journal.pone.0163477

**Published:** 2016-10-03

**Authors:** Jesse Yli-Huumo, Deokyoon Ko, Sujin Choi, Sooyong Park, Kari Smolander

**Affiliations:** 1 Dept. of Innovation and Software, Lappeenranta University of Technology, Lappeenranta, Finland; 2 Dept. of Computer Science & Engineering, Sogang University, Seoul, South Korea; 3 Dept. of Computer Science, Aalto University, Helsinki, Finland; 4 Sogang Institute of Advanced Technology, Sogang University, Seoul, South Korea; West Virginia University, UNITED STATES

## Abstract

Blockchain is a decentralized transaction and data management technology developed first for Bitcoin cryptocurrency. The interest in Blockchain technology has been increasing since the idea was coined in 2008. The reason for the interest in Blockchain is its central attributes that provide security, anonymity and data integrity without any third party organization in control of the transactions, and therefore it creates interesting research areas, especially from the perspective of technical challenges and limitations. In this research, we have conducted a systematic mapping study with the goal of collecting all relevant research on Blockchain technology. Our objective is to understand the current research topics, challenges and future directions regarding Blockchain technology from the technical perspective. We have extracted 41 primary papers from scientific databases. The results show that focus in over 80% of the papers is on Bitcoin system and less than 20% deals with other Blockchain applications including e.g. smart contracts and licensing. The majority of research is focusing on revealing and improving limitations of Blockchain from privacy and security perspectives, but many of the proposed solutions lack concrete evaluation on their effectiveness. Many other Blockchain scalability related challenges including throughput and latency have been left unstudied. On the basis of this study, recommendations on future research directions are provided for researchers.

## Introduction

Currency transactions between persons or companies are often centralized and controlled by a third party organization. Making a digital payment or currency transfer requires a bank or credit card provider as a middleman to complete the transaction. In addition, a transaction causes a fee from a bank or a credit card company. The same process applies also in several other domains, such as games, music, software etc. The transaction system is typically centralized, and all data and information are controlled and managed by a third party organization, rather than the two principal entities involved in the transaction. Blockchain technology has been developed to solve this issue. The goal of Blockchain technology is to create a decentralized environment where no third party is in control of the transactions and data.

Blockchain is a distributed database solution that maintains a continuously growing list of data records that are confirmed by the nodes participating in it. The data is recorded in a public ledger, including information of every transaction ever completed. Blockchain is a decentralized solution which does not require any third party organization in the middle. The information about every transaction ever completed in Blockchain is shared and available to all nodes. This attribute makes the system more transparent than centralized transactions involving a third party. In addition, the nodes in Blockchain are all anonymous, which makes it more secure for other nodes to confirm the transactions. Bitcoin was the first application that introduced Blockchain technology. Bitcoin created a decentralized environment for cryptocurrency, where the participants can buy and exchange goods with digital money.

However, even though Blockchain seems to be a suitable solution for conducting transactions by using cryptocurrencies, it has still some technical challenges and limitations that need to be studied and addressed. High integrity of transactions and security, as well as privacy of nodes are needed to prevent attacks and attempts to disturb transactions in Blockchain [1]. In addition, confirming transactions in the Blockchain requires a computational power.

It is important to identify what topics have been already studied and addressed in Blockchain and what are currently the biggest challenges and limitations that need further studies. To address these questions, we decided to use a systematic mapping study process [2] to identify relevant papers related to Blockchain. In the systematic mapping study, we applied a well-designed research protocol to search for material in scientific databases. The produced map of current research on Blockchain will help other researchers and practitioners in identifying possible research areas and questions for future research.

Although cryptocurrencies are also a business and management topic, we decided to narrow down the research topic to the technical perspective of Blockchain. Our objective was to find and map all papers with technical viewpoints on Blockchain. We were interested in finding Blockchain research topics related to various technical areas, such as security, performance, data integrity, privacy, and scalability.

The rest of the paper is organized as follows. Section 2 introduces the background of Blockchain and Bitcoin. In addition, we present some already identified challenges and technical limitations of Blockchain technology. In Section 3, we describe the applied research methodology and the process of collecting relevant research papers. Section 4 presents the results of the gathered papers and extracted data. Section 5 presents the identified classification schemes. Section 6 discusses the study and answers the research questions. Section 7 concludes the paper.

## Background

Blockchain, mostly known as the technology running the Bitcoin cryptocurrency, is a public ledger system maintaining the integrity of transaction data [1]. Blockchain technology was first used when the Bitcoin cryptocurrency was introduced. To this day, Bitcoin is still the most commonly used application using Blockchain technology [3]. Bitcoin is a decentralized digital currency payment system that consists of a public transaction ledger called Blockchain [4]. The essential feature of Bitcoin is the maintainability of the value of the currency without any organization or governmental administration in control. The number of transfers and users in the Bitcoin network is constantly increasing [5]. In addition, the conversions with traditional currencies, e.g. KRW, EUR and USD, occur constantly in currency exchange markets [6][7]. Bitcoin has therefore gained the attention of various communities and is currently the most successful digital currency using Blockchain technology [6].

Bitcoin uses the public key infrastructure (PKI) mechanism [8]. In PKI, the user has one pair of public and private keys. The public key is used in the address of the user Bitcoin wallet, and the private key is for the authentication of the user. The transaction of Bitcoin consists of the public key of the sender, multiple public keys of the receiver, and the value transferred. In about ten minutes, the transaction will be written in a block. This new block is then linked to a previously written block. All blocks, including information about every transaction made, are stored in the disk storage of the users, called nodes. All the nodes store information about all recorded transactions of the Bitcoin network and check the correctness of each new transaction made by using previous blocks. The nodes are rewarded by checking the correctness of transactions. This method is called mining, and it is confirmed with Proof-of-Work, which is one of the main concepts of Blockchain technology. When all transactions are successfully confirmed, a consensus exists between all the nodes. The new blocks are linked to previous blocks and all the blocks are aligned in one continuous chain. This chain of blocks is the public ledger technique of Bitcoin, called Blockchain.

Blockchain is the decentralized managing technique of Bitcoin, designed for issuing and transferring money for the users of the Bitcoin currency. This technique can support the public ledger of all Bitcoin transactions that have ever been executed, without any control of a third party organization [1]. The advantage of Blockchain is that the public ledger cannot be modified or deleted after the data has been approved by all nodes. This is why Blockchain is well-known of its data integrity and security characteristics. Blockchain technology can also be applied to other types of uses. It can for example create an environment for digital contracts and peer-to-peer data sharing in a cloud service [1]. The strong point of Blockchain technique, data integrity, is the reason why its use extends also to other services and applications.

Blockchain technology has also some technical challenges and limitations that have been identified. Swan [1] presents seven technical challenges and limitations for the adaptation of Blockchain technology in the future:

- **Throughput**: The potential throughput of issues in the Bitcoin network is currently maximized to 7tps (transactions per second). Other transaction processing networks are VISA (2,000tps) and Twitter (5,000tps). When the frequency of transactions in Blockchain increases to similar levels, the throughput of the Blockchain network needs to be improved.
- **Latency**: To create sufficient security for a Bitcoin transaction block, it takes currently roughly 10 minutes to complete one transaction. To achieve efficiency in security, more time has to be spent on a block, because it has to outweigh the cost of double spending attacks. Double-spending is the result of successful spending of money more than once [9]. Bitcoin protects against double spending by verifying each transaction added to the block chain, to ensure that the inputs for the transaction have not been spent previously [9]. This makes latency a big issue in Blockchain currently. Making a block and confirming the transaction should happen in seconds, while maintaining security. To complete a transaction e.g. in VISA takes only a few seconds, which is a huge advantage compared to Blockchain.
- **Size and bandwidth**: At the moment, the size of a Blockchain in the Bitcoin network is over 50,000MB (February 2016). When the throughput increases to the levels of VISA, Blockchain could grow 214PB in each year. The Bitcoin community assumes that the size of one block is 1MB, and a block is created every ten minutes [10]. Therefore, there is a limitation in the number of transactions that can be handled (on average 500 transaction in one block) [11]. If the Blockchain needs to control more transactions, the size and bandwidth issues have to be solved.
- **Security**: The current Blockchain has a possibility of a 51% attack. In a 51% attack a single entity would have full control of the majority of the network’s mining hash-rate and would be able to manipulate Blockchain. To overcome this issue, more research on security is necessary.
- **Wasted resources**: Mining Bitcoin wastes huge amounts of energy ($15million/day). The waste in Bitcoin is caused by the Proof-of-Work effort. There are some alternatives in industry fields, such as proof-of-stake. With Proof-of-Work, the probability of mining a block depends on the work done by the miner [12]. However, in Proof-of-Stake, the resource that is compared is the amount of Bitcoin a miner holds [12]. For example, someone holding 1% of the Bitcoin can mine 1% of the “Proof-of-Stake blocks” [12]. The issue with wasted resources needs to be solved to have more efficient mining in Blockchain.
- **Usability**: The Bitcoin API for developing services is difficult to use. There is a need to develop a more developer-friendly API for Blockchain. This could resemble REST APIs.
- **Versioning, hard forks, multiple chains**: A small chain that consists of a small number of nodes has a higher possibility of a 51% attack. Another issue emerges when chains are split for administrative or versioning purposes.

Overall, Blockchain as a technology has the potential to change the way how transactions are conducted in everyday life. In addition, the applications of Blockchain are not limited to cryptocurrencies, but the technology could be possibly applied in various environments where some forms of transactions are done. The research on the possibilities of Blockchain in applications is certainly an interesting area for future research, but at the moment Blockchain suffers from technical limitations and challenges. Anonymity, data integrity and security attributes set a lot of interesting challenges and questions that need to be solved and assessed with high quality research. Scalability is also an issue that needs to be solved for future needs. Therefore, to identify and understand the current status of research conducted on Blockchain, it is important to gather all relevant research. It is then possible to evaluate what challenges and questions have been tackled and answered, and what are the most problematic issues in Blockchain at the moment.

## Research methodology

**Fig 1:**
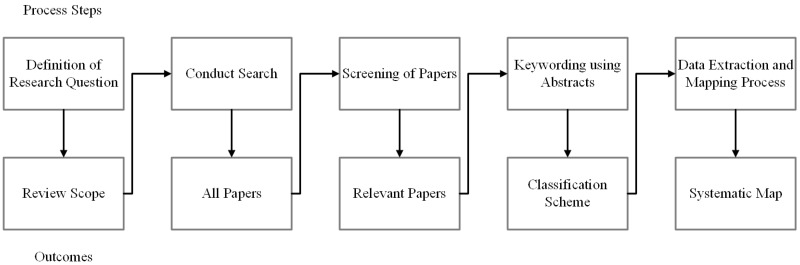
The systematic mapping process.

Systematic mapping study was selected as the research methodology for this study. The goal of a systematic mapping study is to provide an overview of a research area, to establish if research evidence exists, and quantify the amount of evidence [2]. In this study we follow the systematic mapping process described by Petersen et al. [13]. We also use guidelines for a systematic literature review described by Kitchenham and Charters [2] to search for relevant papers. We chose the systematic mapping process as our research methodology because our goal was to explore the existing studies related to Blockchain technology. The results of the mapping study would help us to identify and map research areas related to Blockchain technology and possible research gaps. The process for the systematic mapping study is presented in Fig 1, and consists of five process steps and outcomes. The Prisma Checklist is provided in S1 Checklist.

### Definition of research questions

The first stage of the systematic mapping process is the definition of the research questions. The goal of this study was to provide an overview of the current research on Blockchain technology. Therefore, we defined four research questions:

- **RQ1: What research topics have been addressed in current research on Blockchain?**
  The main research question of this mapping study is to understand the current research topics on Blockchain. By collecting all the relevant papers from scientific databases, we would be able to create an overall understanding of Blockchain research and map the current research areas. Mapping the current research done on Blockchain technology will help other researchers and practitioners to gain better understanding on the current research topics, which will help to take the research on Blockchain even further.
- **RQ2: What applications have been developed with and for Blockchain technology?**
  Blockchain is mostly known for its relation to Bitcoin cryptocurrency. Bitcoin uses Blockchain technology in currency transactions. However, Bitcoin cryptocurrency is not the only solution that uses Blockchain technology. Therefore, it is important to find the current applications developed by using Blockchain technology. Identifying other applications can help to understand other directions and ways to use Blockchain.
- **RQ3: What are the current research gaps in Blockchain research?**
  A systematic mapping of research enables understanding the current research gaps. The identification of research gaps will help other researchers and practitioners to focus their research on areas that require more research. Finding research gaps will help to understand and find unanswered research questions in current Blockchain technology.
- **RQ4: What are the future research directions for Blockchain?**
  Understanding the potential future research directions for Blockchain technology is a consequence of RQ1-RQ3. Answering this research question is beneficial when deciding where the research on Blockchain technology should be directed and what issues need to be solved.

### Conducting the search

The second stage of a mapping study is to search for all the relevant scientific papers on the research topic. A search protocol defines the methods that will be used to undertake a specific systematic literature search. A pre-defined protocol is needed to reduce the possibility of researcher bias [2].

We created a search protocol that we used for scientific databases to gather all the papers relevant for our research topic. The terms used in the search string were chosen after pilot searches, where we tested possible keywords. After the pilot search we decided to use only the term Blockchain as the search string, even though Bitcoin could also have been a possible one. However, in the pilot search we used also Bitcoin as a search term, but we identified a huge number of papers that were related to economic topics in cryptocurrencies, rather than technological aspects of Blockchain technology. Therefore, since our goal in this mapping study process was to find and map the papers related to technical aspects of Blockchain technology, we decided to drop the term Bitcoin. We believe that by using only the term Blockchain as the search string, the majority of Bitcoin-related papers with a technical perspective on Blockchain were still included. In addition, it seemed that if a Bitcoin-related paper did not have the term Blockchain anywhere in its meta-data, the paper was related to the economics of a cryptocurrency.

After designing and testing the search protocol, we chose the scientific databases for the searches. We decided to concentrate on peer-reviewed, high quality papers published in conferences, workshops, symposiums, books and journals related to the research topic. We used six scientific databases for paper retrieval. The chosen databases were (1) IEEE Xplore, (2) ACM Digital Library, (3) Springer Link, (4) ScienceDirect, (5) Ebsco, and (6) PLOS One. We decided not to use grey literature e.g. from Google searches, and kept scientific peer review as the criterion.

### Screening of relevant papers

Because all papers in the searchers were not necessarily related to the research questions, they needed to be assessed for their actual relevance [2]. After using the search protocol in the scientific databases, the next stage was the screening of papers. For screening the relevant papers, we used a process inspired by Dybåand Dingsøyr [14]. At the first screening phase, we screened the papers based on their titles and excluded studies that were not relevant to the research topic. For example, the search protocol returned papers related to Blockchain in other scientific fields, which had different meaning than the Blockchain technology used in computer science. These papers were clearly out of the scope of this mapping study, which was a valid reason to exclude them. However, in some cases it was difficult to determine the relevancy of the paper on the basis of the title of the paper. In these situations, we passed the paper through to the next stage for further reading. In the second phase, the authors read the abstracts of every paper that passed the previous phase. In addition, we used specific inclusion and exclusion criteria to screen each paper. We decided to exclude the following types of papers: (1) papers without full text availability, (2) papers where the main language was not English, (3) papers that had some other meaning than Blockchain used in computer science, (4) papers that were duplicates, and (5) papers that were posters. When a paper passed all the five exclusion criteria, and after reading the abstract it was considered as focusing on Blockchain, we decided to include it in the next screening stage.

### Keywording on the basis of the abstract

**Fig 2:**
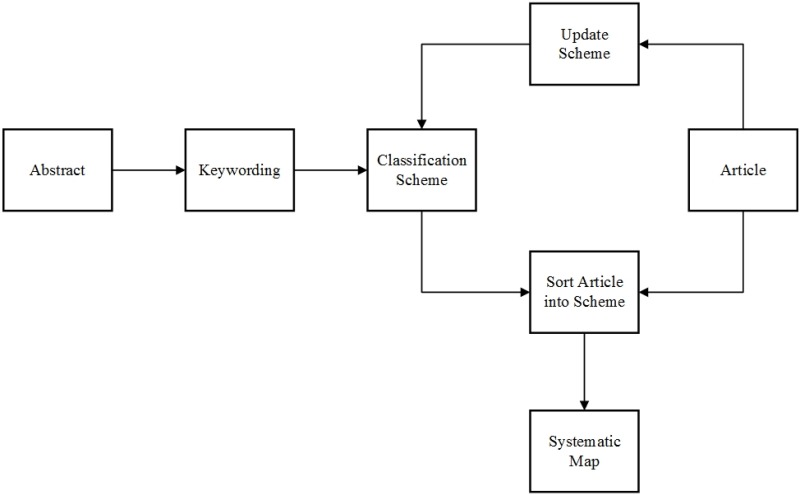
Building the classification scheme.

The next stage in a mapping study process after finding the relevant papers through abstracts is keywording. For this stage, we used the process defined by Petersen et al. [13] (Fig 2). Keywording was done in two steps. In the first step we read the abstract and identified keywords and concepts that reflected the contribution of the paper [13]. The second step was to develop a higher level of understanding based on these keywords [13]. We used the keywords to cluster and form categories for the mapping of the studies. After the categories had been clustered, we read all the selected papers. After the reading we also updated the categories or created new ones, if the paper revealed something new. This resulted in a systematic map of clustered categories formed from all the relevant papers on the research topic.

### Data extraction and mapping process

A data extraction form (Table 1) was designed to collect the information needed to address the research questions of this mapping study [2]. Data items DI0 to DI6 gathered basic information of the papers. These items included e.g. the title of the paper, the name(s) of the author(s), the country of the author(s), and publication type/place. The rest of the data items (DI7-DI10) were gathered after reading the papers. These data items included e.g. study goals and major findings of each paper. We collected the extracted data items to Excel, which helped us to organize and analyze the data.

**Table 1:** Data extraction items.

#	Data item	Description
DI0	Study identifier	Study id (e.g. ID01)
DI1	Title	Title of the paper
DI2	Authors	Name of the author(s)
DI3	Country	Country of authors
DI4	Publication info	Name of the publication place
DI5	Publication type	Type of publication (e.g. conference/workshop/journal)
DI6	Publication source	Academia / Industry
DI7	Abstract	Abstract of the paper
DI8	Study aim	Aim of the paper
DI9	Research question/goal	Research questions/goals defined for the paper
DI10	Study findings	Major findings of study

## Basic information of the papers

In this section, the search and selection results of the systematic mapping study are presented. Out of the extracted data items (Table 1), this section reports on data items DI0-DI6.

### Search and selection results

**Fig 3:**
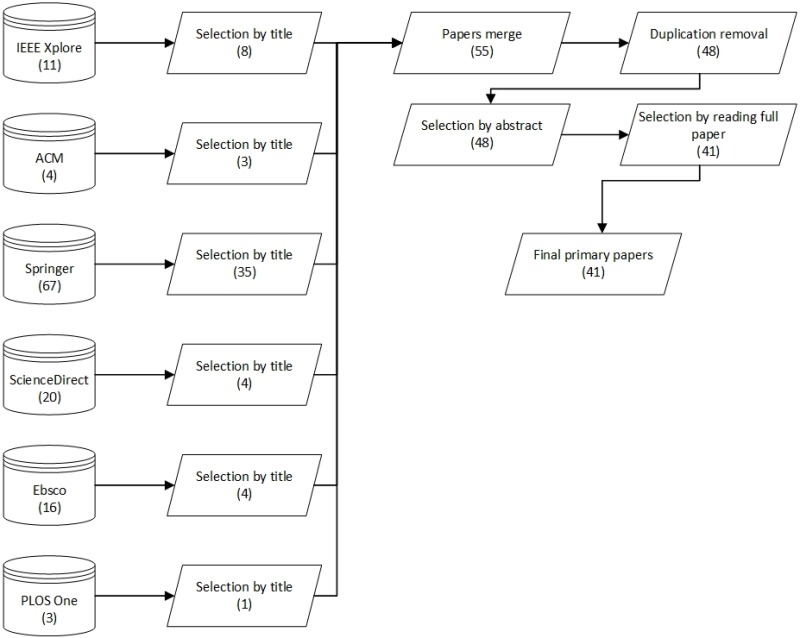
Search and selection process of the papers.

The search and selection results are presented in Fig 3. The PRISMA flow diagram is also provided in S1 Diagram. 121 papers were initially retrieved when the designed search protocol was applied to the selected scientific databases. The first inclusion and exclusion round was based on the titles of the retrieved papers. All the paper titles were examined by two authors, which led to the selection of 55 papers. The reason for the high number of excluded papers (66) was that they were not related to the research topic. For example, many excluded papers discussed the business perspective of Bitcoin, and therefore they did not belong to our study. We also retrieved multiple papers related to other scientific areas, such as chemistry and mathematics, where the keyword Blockchain had another meaning than the technology used in computer science.

After the selection of 55 papers, we removed duplicates and used the next round exclusion and inclusion criteria defined in section 3.3. This round resulted in the selection of 48 papers. After this, three authors read the abstracts of all the selected papers. This did not result in the exclusion of any papers, however. Based on the abstracts, all the selected papers had a topic related to Blockchain with a technical viewpoint.

However, we decided to pass some unclear papers to the next selection round for more in-depth analysis. In the last stage of paper selection, three authors read all the papers. This resulted in the selection of 41 papers, which we included in this study as primary papers. Three papers were dropped due to their focus on the economic perspective of Blockchain and Bitcoin. Additional four papers were excluded for being only reports describing Blockchain and how it works without providing any actual new research findings or evidence. The full list of the selected papers with the extracted data items is presented in S1 Table.

### Publication year, source and geographic distribution

**Fig 4:**
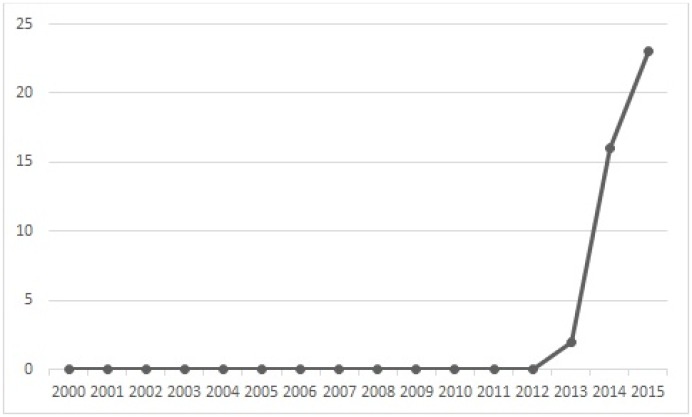
Publication year of the selected primary papers.

Fig 4 shows the publication year distribution of the selected primary papers. Interestingly, all the selected papers were published after the year 2012. This shows that Blockchain as a research area is a very recent and new one. When looking at the publication year distribution more closely, out of all the selected papers, 2 papers (5%) were published in 2013, 16 papers (39%) in 2014 and 23 papers (56%) in 2015. This shows an increasing number of publications each year, which suggests also a growing interest in Blockchain technology. This is not a surprise, because the idea of Blockchain and Bitcoin was first coined only in 2008 [4].

**Fig 5:**
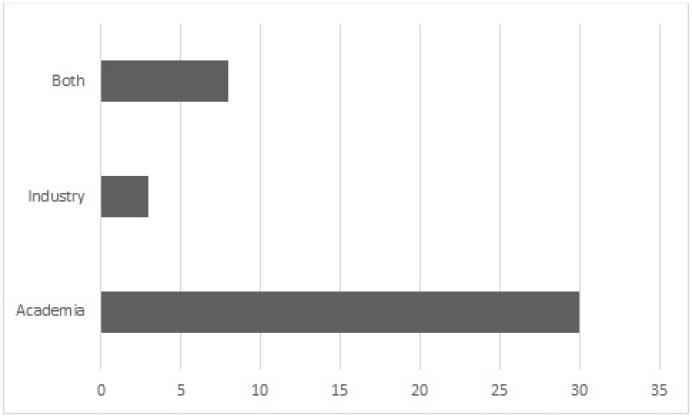
Source of the selected primary papers.

Fig 5 shows the source of each selected primary paper. The possible sources for a paper are the academia, industry, or both. Our results showed that 30 papers (73.1%) were published by an academic source and only 3 papers (7.3%) were published by an industry source. In 8 papers (19.5%), the authors were from both academia and industry. It is, however, highly possible that most of the papers published by the industry are not included in scientific databases. Most industry papers can be found as white papers and are not often published in peer-reviewed conferences or journals.

**Fig 6:**
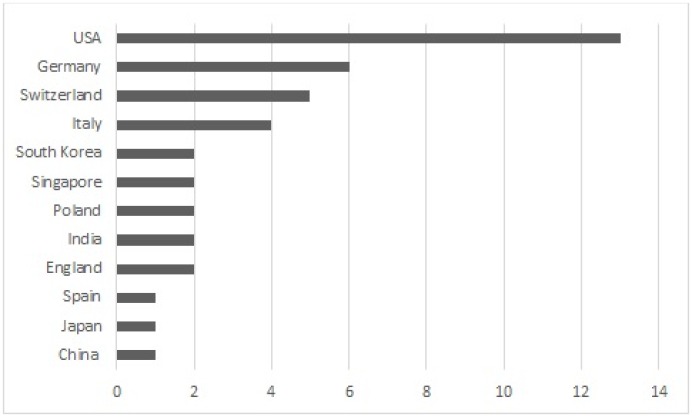
Geographic distribution of the selected primary papers.

The geographical distribution of the selected papers is shown in Fig 6. The largest number of papers (13, 31%) were published by universities or companies in the USA. After this, the two most common publication countries were Germany with 6 papers (14.6%) and Switzerland with 5 papers (12.2%). The rest of the countries had four or less papers published. The geographical distribution of the selected primary papers shows that Blockchain technology has gathered research interest around the world.

### Publication type and channel

**Fig 7:**
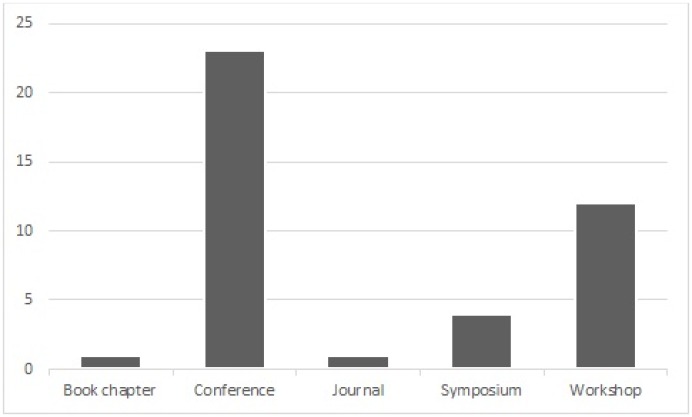
Publication type.

Fig 7 shows the publication type of the selected papers. Publication type means the channel where the paper has been published. The publication types included in this mapping study were conference, journal, workshop, symposium, and book chapter. Most of the papers were published in conferences (23) (56%) and workshops (12) (29.2%). The rest of the papers were published in symposiums (4) (9.7%), as a book chapter (1) (2.4%), or in a journal (1) (2.4%). In addition, Table 2 shows the publication channel of each selected paper. Most papers were published in conferences and workshops in the International Conference on Financial Cryptography and Data Security (FC) (13) (31.7%). 3 or less of the selected papers had used other publication channels.

**Table 2:** Publication channels.

Canadian Conference on Electrical and Computer Engineering (CCECE)	[15]
International Conference on Information Systems Security (ICISS)	[16]
International Workshop on Security and Trust Management (STM)	[17][18]
International Conference on Applied Cryptography and Network Security (ACNS)	[19]
International Conference on Software Engineering, Artificial Intelligence, Networking and Parallel/Distributed Computing (SNPD)	[20]
International Conference on Passive and Active Measurement (PAM)	[21]
International Conference on Intelligence in Next Generation Networks (ICIN)	[22]
International Conference on Financial Cryptography and Data Security (FC)	[23][24][25][26][27][28][29][30][31][32][33][34][35]
European Symposium on Research in Computer Security (ESORICS)	[36][37][38]
International Conference on the Theory and Applications of Cryptographic Techniques (EUROCRYPT)	[39]
International Cryptology Conference (CRYPTO)	[40]
International Conference on Trust & Trustworthy Computing (TRUST)	[41][42]
International Conference on Network and System Security (NSS)	[43][44]
Book: Programming Languages with Applications to Biology and Security	[45]
ACM Conference on Computer and Communications Security (ACM CCS)	[46]
ACM Conference on Data and Application Security and Privacy (CODASPY)	[47]
International Conference on Computational Science and Applications (ICCSA)	[48]
eCrime Researchers Summit (eCRS)	[49]
International Conference on Big Data and Cloud Computing (BDCloud)	[50]
IEEE Symposium on Visualization for Cyber Security (VizSec)	[51]
International Conference on Peer-to-Peer Computing (P2P)	[52]
International Workshop on Secure Peer-to-Peer Intelligent Networks & Systems (SPINS)	[53]
IEEE Micro Magazine	[54]
International Workshop on Data Privacy Management (DPM)	[6]

## Classification of the relevant papers

In this section, the classification of the selected primary papers is presented, including extracted data items DI7-DI10 (Table 1). After reading all the selected papers and creating classifications based on the findings, we identified that a majority of the papers were related to the technical challenges and limitations presented by Swan [1]. Therefore, we decided to use these challenges and limitations for the classification to map the existing research on Blockchain. The challenges and limitations presented by Swan are throughput, latency, size and bandwidth, security, wasted resources, usability, versioning, hard forks, and multiple chains. In addition, we identified a new classification type, privacy. Privacy in an essential attribute in the Blockchain environment, because of its anonymity characteristic. In addition, we also used the class others to map papers that were not related to any of the classes mentioned above.

**Fig 8:**
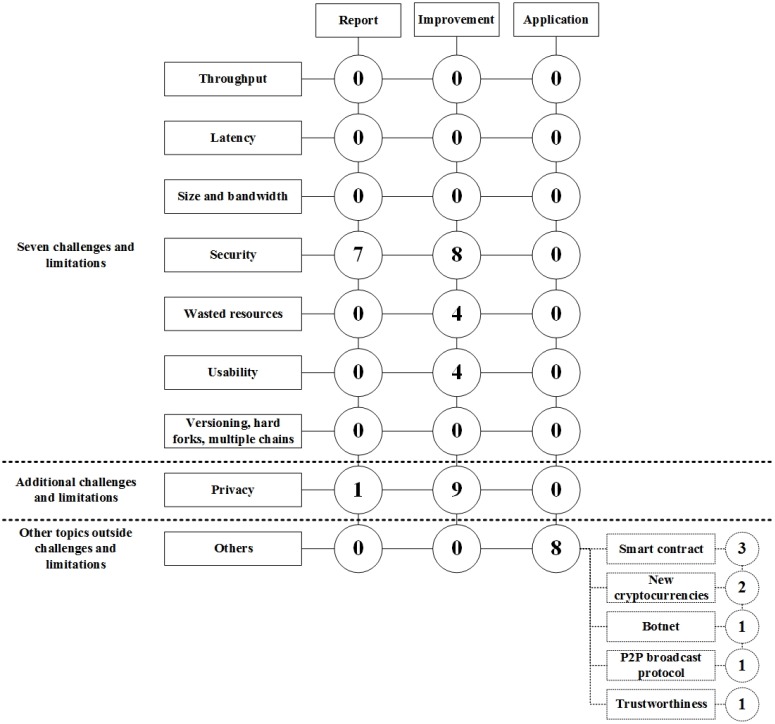
Classification of the relevant papers.

We also identified that there were three different paper types for each class, Blockchain report, Blockchain improvement and Blockchain application. A Blockchain report includes papers that report previously identified solutions and ideas in Blockchain and Bitcoin. A Blockchain improvement includes papers that suggest new solutions and improvements to the current Blockchain or Bitcoin technology. A Blockchain application includes papers that present an application based on Blockchain technology. The final map of this study is presented in Fig 8.

**Fig 9:**
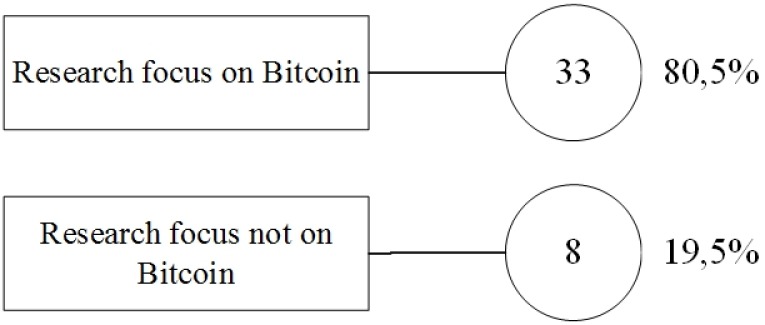
Bitcoin related research.

We also decided to examine the papers based on their relation to Bitcoin (Fig 9), because it is considered so far the most important and commonly used solution based on Blockchain technology. As expected, a great number of papers were related to Bitcoin, rather than other applications. In 33 (80.5%) of the selected papers, the research was conducted in the Bitcoin environment. We found only 8 papers (19.5%) that did focus on Bitcoin, but on other applications using Blockchain technology.

**Fig 10:**
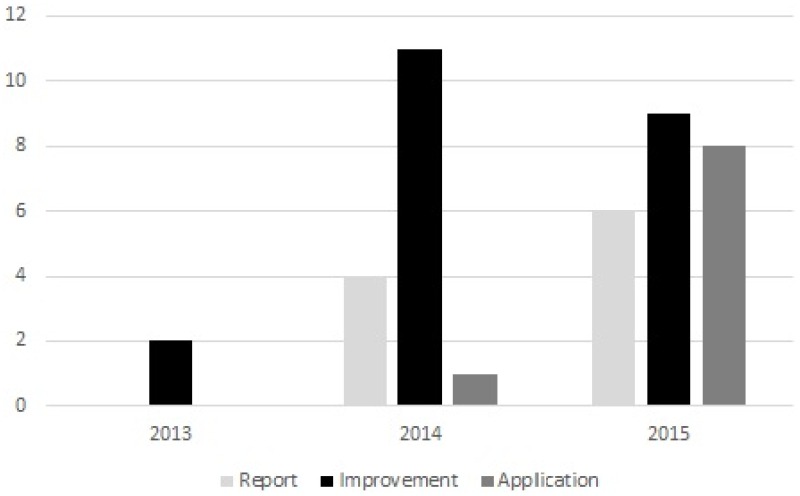
Paper types by year.

We also made a comparison between the paper type (Blockchain report, Blockchain improvement, and Blockchain application) and the publication year. The comparison is shown in Fig 10. The figure shows an increasing number of papers in both report and application categories over the three years. Improvement papers had a significant increase in 2014, but a decrease in 2015.

### Security

Security was the one of the major research topics in the selected primary papers. 14 out of the 41 papers (34%) were related to challenges and limitations in Blockchain and Bitcoin security. We identified various topics in security, including trends and impacts of security incidents, 51% attack, data malleability problems, and authentication and cryptography issues.

**Trends and impacts of security incidents**: With the increasing use of Bitcoin as a way to conduct payments and transfers, security incidents and their impact on the economic losses of Bitcoin users have increased. Some of the identified papers presented security incidents that had occurred in the Bitcoin network, such as economic losses by several Bitcoin scams and distributed denial-of-service (DDoS) attacks on exchanges and mining pools. Vasek et al. [33] investigated four types of Bitcoin scams (Ponzi scams, mining scams, scam wallet and fraudulent exchanges) by tracking online forums and voluntary vigilantes. The authors noted that $11 million had been contributed to scams by 13000 victims in Bitcoin from September 2013 to September 2014. Lim et al. [48] analyzed the trend of security breaches in Bitcoin and their countermeasures. According to the authors, all possible types of security breaches had occurred, including DDoS attacks, private account hacking using Trojan horses, or viruses from ads. The authors introduce some security countermeasures for individual users and safe Bitcoin transactions (e.g. a hardware wallet and a hardware authentication device). Vasek et al. [27] present evidence on DDoS attacks in the Bitcoin network using DDoS-related posts in the popular Bitcointalk.org forum. The authors figured out that the most targeted service category was the use of anti-DDoS protection, influencing factors such as the mining pool size. The major findings of the study were that the most often targeted service was currency exchange (41%), followed by mining pools (38%). According to the paper, 54% of the services that had experienced DDoS attacks had anti-DDoS protection, although it was not certain whether they had the protection on at the time of attack. In addition, of the services that had not yet experienced a DDoS attack, only 15% had anti-DDoS protection. The paper concludes that over 60% of large mining pools have suffered DDoS attacks, compared to 17% of small pools.

**51% Attack**: The Blockchain mechanism is designed with the assumption that honest nodes control the network [4]. If attacker nodes collectively control more computational power than the good ones, the network is vulnerable to the so called 51% Attack. Beikverdi et al. [20] argue that although the Bitcoin itself is designed as a fully decentralized network, market-based centralization of mining power by a few large mining pools increase the risk of a 51% Attack. Their study shows that the centralization factor of Bitcoin has been continuously increasing from 2011 (0.26) to 2014 (0.33). In this context, 0 means purely decentralized and 1 means fully centralized. Moreover, there are studies claiming that the 1/2 assumption of computational power is not enough for security. Garay et al. [39] propose applications built on the core of the Bitcoin protocol focusing on the Byzantine agreement (BA), which is the fundamental scientific problem for decentralized transaction agreement in the Bitcoin network. The suggested application presents a simple BA protocol with the assumption that the adversary’s hashing power is bounded by 1/3. Eyal and Sier [30] introduce a Selfish Mine attack where colluding miners obtain a revenue larger than a fair share by keeping their discovered blocks private. The authors propose a protocol modification which commands less than 1/4 of the total computation power.

The more recent Blockchain-based systems, such as Ethereum, allow users to specify scripts in transactions and contracts to support applications beyond simple cash transactions. In this case, the required computational resources for verification could be larger, depending on the user-specified script size. Luu et al. [46] present a security attack called the verifier’s dilemma, which drives rational miners to skip verification where the verifying transactions require significant computational resources in Bitcoin and especially in Ethereum. The authors formalize a consensus model to give incentives to miners by limiting the amount of work required to verify a block.

Armknecht et al.[42] explain how to support security and privacy in the Ripple system, which is one of the consensus-based distributed payment protocols. The paper discusses the basic difference between the protocol of Ripple and Bitcoin-focused Blockchain fork. A fork can occur if two conflicting ledgers get a clear majority of votes, and could lead to double spending attacks. According to Decker and Wattenhofer [52], the propagation delay in the Bitcoin network is the primary cause for Blockchain forks and inconsistencies among replicas, which was done by analyzing Blockchain synchronization mechanism.

**Data malleability problems**: Data integrity is an essential issue in the Blockchain environment. It is necessary that when data gets sent and verified, it has not been altered or tampered with. We found two studies related to data integrity that studied malleability attacks in Blockchain. Malleability describes the fact that the signatures that prove the ownership of Bitcoin being transferred in a transaction do not provide any integrity guarantee for the signatures themselves [36]. Therefore, in a malleability attack an attacker intercepts, modifies, and rebroadcasts a transaction, causing the transaction issuer to believe that the original transaction was not confirmed [36].

Decker & Wattenhoffer [36] studied transaction malleability in Bitcoin environment and used a real-life case as an example. According to the paper, the transaction malleability problem is real and should be considered when implementing Bitcoin clients. Andrychowicz et al. [31] made a similar study by conducting practical experiments which presented a high possibility of a malleability attack and its impact. In their study, the malleability attack caused incorrect balance computing, application crashes, and a deadlock which stopped new transactions in several well-known Bitcoin wallets. The paper suggests a deposit protocol with a timed commitment scheme to enable a malleability-resilient refund transaction as a solution to the malleability problem.

**Authentication and cryptography issues**: In Bitcoin, the private key is the major authentication element. Authentication in cryptocurrency controls self-certification. There have been some incidents with authentication. For example, there is the well-known case in Mt.Gox, where a Bitcoin wallet company was attacked. In the attack, Mt.Goxs storage that included private keys of their customer was stolen. This incident has motivated some studies in strengthening authentication in Bitcoin. In addition to the Mt.Gox case, Bos et al. [26] state that the use of elliptic curve cryptography (ECC), which is used to derive Bitcoin addresses to users, is insufficient and does not have the required randomness.

We identified a number of papers that had the goal to address the issues in the Bitcoin authentication process. Bamert et al. [18] suggest a Bitcoin hardware token, the BlueWallet. The device communicates by using Bluetooth Low Energy, and is able to secure and sign Bitcoin transactions. Ateniese et al. [19] propose a certification system for Bitcoin that offers an opt-in guarantee to send and receive Bitcoins only to/ from certified users, and control of the creation of Bitcoins addresses by trusted authorities. According to the paper, this approach improves the trustworthiness of real-world entities into the system, which mitigates the existing reservations to the adoption of Bitcoin as a legitimate currency. Mann et al. [17] suggest two-factor authentication for a Bitcoin wallet. The authors used a smart phone as the second authentication factor. The solution can be used with hardware already available to most users, and the user experience/interface has similarities to the existing online banking authentication methods.

### Wasted resources

The energy efficiency problem is not handled in the computer engineering field at the moment. However, in special domains like mobile cloud computing, it might be one of the major issues in the future [55]. Mining Bitcoins requires a high amount of energy to compute and verify transactions securely and with trustworthiness [1]. However, for the efficiency of mining and Proof-of-Work, it is important to decrease the amount of wasted resources.

We identified some papers related to the wasted resource problems in Bitcoin. Wang and Liu [21] present the evolution of Bitcoin miners in terms of volume of solo and pool miners and their productivity. In the early stages, the computation power was evenly distributed among the solo miners. As the Bitcoin network evolved, the computation power of some pool miners increased. The study notes that all miners play a zero-sum-computation race game: *each miner increases their computation power, and then the total computation power in the network increases; consequently the system increases the difficulty value to maintain a steady Bitcoin creation speed, which in turn reduces the Bitcoin mining rate of individual miners* [21].

We also identified some papers that proposed solutions for the wasted resources problem in Blockchain and Bitcoin. Wang and Liu [21] suggest an economic model for getting high economic returns in consideration of the use of mining hardware with high computation-over-power efficiency and electricity price. Paul et al. [16] have calculated and show how a new scheme can lead to an energy-efficient Bitcoin. The authors modified the present block header by introducing some extra bytes to utilize the timestamp more effectively. The suggested scheme uses less computing power, and thus the mining is more environment-friendly. Anish [15] proposes methods of achieving contextually higher speeds of Bitcoin mining, involving simultaneous usage of CPUs and GPUs in individual machines in mining pools. The results presented in the paper show how standard hardware miners in large mining pools could quite significantly add to the overall hash rate. Barkatullah et al. [54] describe the architecture and implementation details of a CoinTerras first-generation Bitcoin mining processor, Goldstrike 1, and how this processor was used to design a Bitcoin mining machine called Terraminer IV, especially about how high power density issues were solved and energy efficiency increased.

### Usability

The original definition of the challenges and limitations in the usability of Blockchain by Swan [1] describes Bitcoin API as hard and difficult to use. This definition can be viewed mainly from the developer’s perspective, where Bitcoin API is hard to implement and use in and with other services and applications. We did not find any papers related to the usability issue from the software developer’s perspective. However, we found several papers that considered the usability of Bitcoin from the cryptocurrency user’s perspective. Therefore, we decided to expand the original definition of Blockchain usability to take usability into account also from the point of view of the cryptocurrency user.

An important factor in Blockchain usability from the user’s perspective is the ability to analyze Blockchain. In Blockchain, new blocks are created constantly and confirmed by miners, which creates an interesting environment of transaction flows. It is therefore essential to have supporting tools to help users analyze the whole Blockchain network to improve the usability. We found applications that had been developed for this purpose. BitConeView [51] is a system for the visual analysis of Bitcoin flows in Blockchain. BitIodine [23] parses Blockchain, clusters addresses that are likely to belong to the same user or group of users, classifies such users and labels them, and finally visualizes the complex information extracted from the Bitcoin network. Both these systems were tested successfully with experiments and cases, and showed effectiveness in analyzing and detecting patterns in the Bitcoin network. These systems can help also in improving security and privacy -related issues.

Bankruptcy and the closure of Bitcoin exchanges can cause economical damage to the customers [38]. Decker et al. [38] propose an audit software to improve usability in Bitcoin exchanges. The goal of the software is to prove the exchange participants’ solvency without publishing important information. In addition, Vandervort [25] discusses the link between a buyer and a seller with a layer of limited anonymity, thus preventing buyers from finding or validating information in Bitcoin. The paper presents three different models by which a reputation/rating system could be implemented in conjunction with Bitcoin transactions, and considers the pros and cons of each. Improving these aspects of exchanges done in the Bitcoin network can improve the usability by providing additional information for the users making the transactions.

### Throughput, latency, size and bandwidth, and versioning, hard forks, multiple chains

Interestingly, we did not identify any papers that were related to other technical challenges and limitations, such as throughput, latency, size and bandwidth, versioning, hard forks, and multiple chains.

### Privacy

In a Blockchain network, a distributed consensus network without a trusted party, all the transactions are transparent and announced to the public. Therefore, privacy in Blockchain is maintained by breaking the flow of information. The public can see all transactions, but without information linking the transaction to identities [4]. For this security model, 10 studies out of 41 (24%) proposed privacy issues and countermeasures to increase anonymity in Blockchain.

Meiklejohn and Orlandi [32] present a definitional framework of anonymity focusing on the ownership of the coin. There are also studies that show experimental evidence on the lack of anonymity in the Bitcoin network. Koshy et al. [35] analyzed a traffic pattern in Bitcoin and conclude that some subset of Bitcoin addresses can be mapped to an IP address simply by observing the transaction relay traffic. Feld et al. [53] introduce a framework to traverse the Bitcoin network and generate statistics based on that. By using the tool, the authors figured out that an average peer-list contains addresses that mostly reside in the own autonomous systems of the peers. Taking this information into account, the authors claim that transaction linking could be possible.

Similar to our mapping study, Herrera-Joancomartí [6] provide an exhaustive review of papers on Bitcoin anonymity research. according to the author, very few papers have been published regarding the traffic of Bitcoin that may reveal private information. In order to solve the anonymity reduction, a mix of services has been proposed in some papers. A number of studies have applied a transaction mixing technique to increase privacy. A mixing transaction allows the users to move Bitcoins from one user address to another without a clear trace linking between the addresses. Such transactions can act as a primitive to help improve anonymity when transaction linking becomes more challenging.

Valenta and Rowan [24] have modified the Mixcoin protocol to prevent the mix from learning the input/output address mappings of participating users. The authors propose a system, Blindcoin, which modifies the Mixcoin mixing protocol by using blind signatures and a public append-only log. The log makes it possible for a third party to verify the validity of accusations when blind signatures are used. Ziegeldorf et al. [47] present CoinParty, a decentralized mixing service for Bitcoin based on a combination of decryption mix-nets with threshold signatures. According to the authors, CoinParty is secure against malicious adversaries, and the evaluation of their prototype shows that it scales easily to a great number of participants in real-world network settings. Ruffing et al. [37] propose CoinShuffle, a completely decentralized Bitcoin mixing protocol that allows the users to utilize Bitcoin in a truly anonymous manner. It does not require any (trusted, accountable or untrusted) third party and it is compatible with the current Bitcoin system. CoinShuffle introduces only a small communication overhead for its users, while avoiding additional anonymization fees and minimizing the computation and communication overhead for the rest of the Bitcoin system. Androulaki et al. [41] propose a solution, an extension of ZeroCoin (EZC), to hide transaction value and address balances in Bitcoin for increased privacy. ZeroCoin acts as a temporary currency to impede the traceability of coins, but it does not hide the number of transactions and balances of Bitcoin addresses. The proposed improvements include mixing Bitcoins from various sources before sending them to a destination and enabling payments in the form of EZC without the need to transform them back to Bitcoin. For the effectiveness of mixing techniques in improving anonymity, Möser et al. [49] present analysis results on some available Bitcoin mixing services. The test results showed that linking the input and output transactions was possible in 1 out of 3 tested services. Other than mixing techniques, Saxena et al. [28] suggest use of composite signatures to prevent linking between sending and receiving addresses.

### Smart contracts, new cryptocurrencies, botnet, broadcast protocol, trustworthiness

We also identified other classifications that were not included in the seven technical challenges and limitations defined by Swan [1]. Three of the papers were related to the use of Smart contracts in the Blockchain environment. A smart contract is a solution that utilizes Blockchain technology to create contracts between two or more participants. Similarly to the use of Bitcoin Blockchain, smart contracts are done in a decentralized environment, where contract terms are executed by the Blockchain systemwhen the terms are fulfilled. Bigi et al. [45] introduce a decentralized smart contract protocol inspired by BITHALO and validated the feasibility of the protocol based on the protocols of Bitcoin. The approach is a combination of the game theory and formal models. The authors argue that a decentralized smart contract system can be a promising approach and worthy of being studied and developed further. Wan et al. [43] propose an electronic signing protocol between two parties using the Bitcoin network as a way of providing a time-stamping service. In addition, smart contracts can be possibly used in various environments and industries for different purposes. For example, Kishigami et al. [50] provide a Blockchain-based digital content distribution system and show a prototype of the concept. The idea was presented to one hundred people including creators, content owners and digital content stake holders. The feedback showed that the most impressive point was the decentralized mechanism for Digital Right Management. However, the proposed system has no incentive mechanism for mining calculation, which can make it a challenge to adopt at the moment.

Even though Bitcoin is the most famous and commonly used cryptocurrency adopting Blockchain technology, there has also been research on developing other cryptocurrencies. Zhang and Wen [22] have designed a new generation cryptocoin called IoTcoin, based on the protocol of Bitcoin and Blockchain. In IoT-coin, people can use keys and scripts which are obtained in them to exchange paid sensor data or smart property. IoTcoins can be used to present the ownership of many IoT commodities, such as smart property, paid data and digital controlled energy. Another cryptocurrency has been proposed by Vandervort et al. [29] as a model of a community cryptocurrency with a community fund feature.

We also found three papers that used Blockchain for Botnet networks, a P2P broadcast protocol, and a trustworthiness improvement. Ali et al. [34] ppresent Zombiecoin, which runs in Bitcoin networks and offers a Botnet C&C (command-and-control) mechanism. Botnet networks include a number of computers communicating in an effort to compute representative tasks. However, the weak point for botnet is the C&C infrastructure. The Bitcoin transaction can be used as a communication vehicle. Andrychowicz and Dziembowsk [40] ppresent a formal model for peer-to-peer communication and a Proof-of-Work concept used in Bitcoin, and based on the model, propose a broadcast protocol which is more secure against an adversary with arbitrary computational power. Wilson and Ateniese [44] have adopted the Bitcoin technology to enhance the Pretty Good Privacy (PGP) mechanism. In this mechanism, a Bitcoin address, Bitcoin identity verification transactions, and a Blockchain key server are used to improve the user’s trustworthiness.

### Summary of the identified challenges/limitations and suggested solutions in Blockchain

**Fig 11:**
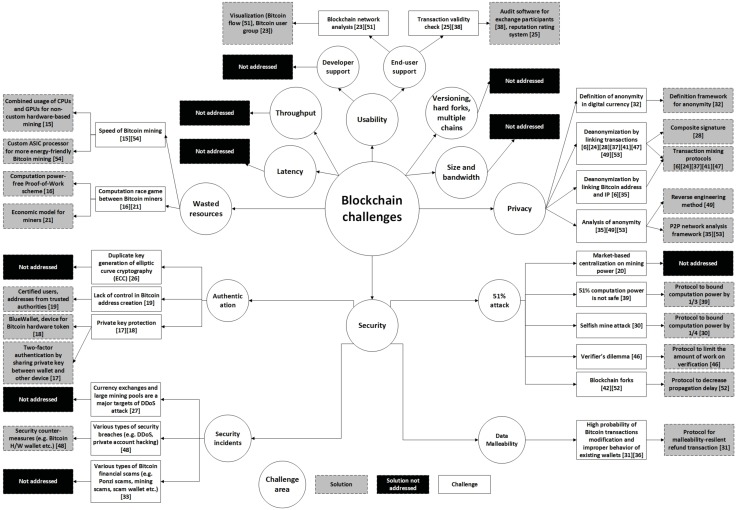
Summary of the identified challenges and solutions of Blockchain.

In Fig 11 we summarize the identified challenges and suggested solutions in Blockchain and Bitcoin.

## Discussion

In this chapter we discuss the results and answer the four main research questions. In addition, at the end of this chapter, we discuss the limitations and validity of the study.

### RQ1: What research topics have been addressed in current research on Blockchain?

The results of this mapping study showed that a majority of the current research on Blockchain is focused on finding and identifying improvements to the current challenges and limitations in Blockchain [1]. A large portion of the research concentrates on security and privacy issues in Blockchain.

The security vulnerability of the Blockchain network and the growing interest in Bitcoin have increased the economic losses of both miners and end users. The identified vulnerabilities include computation power -based attacks, such as the 51% attack, selfish mine attack, transaction data malleability problems, and deanonymization by transaction linking. Although several solutions to address these issues have been presented, many of them are just brief idea suggestions, lacking concrete evaluation of their effectiveness.

The research on other topics in challenges and limitations described by Swan [1], such as wasted resources and usability, was rather limited. We found some research done on computational power and wasted resources in Bitcoin mining, and improvements on the usability of Bitcoin. However, the number of papers was considerably small compared to those on security and privacy issues. Computational power is one of the key attributes in Blockchain, and it requires attention in the research. When Blockchain grows more complex, it also requires more computational power to confirm more blocks. The Proof-of-Work concept is a rather new idea, which is the reason why it has to be studied more, to make sure that it can work in large-scale Blockchain environments.

Interestingly, we did not find many studies on challenges and limitations in latency, size and bandwidth, throughput, versioning, hard forks, and multiple chains. It is surprising that the attention paid to and research done on other challenges and limitations than security and privacy was rather low. We assumed that especially topics like latency, size and bandwidth, and wasted resources would have received more attention in the overall research map. When the size of Blockchain increases, it has a direct impact on all these challenges and limitations in scalability. It is possible that these issues have not been studied a lot because the Blockchain concept is still rather new.

In addition to the identified research topics, the findings in this mapping study showed that a majority of research was conducted in the Bitcoin environment. This was also the original assumption of the authors, considering that Bitcoin is currently the most commonly used and important technology using Blockchain, with the largest user base. However, we were quite surprised that the number of other solutions than Bitco using Blockchain was so low. The results showed that the research outside the Bitcoin environment was mostly focused on smart contracts and other cryptocurrencies, but the research on Bitcoin and its security issues formed the majority.

### RQ2: What applications have been developed with and/to Blockchain technology?

We originally defined a Blockchain application as a solution that has been developed with Blockchain technology. By this definition, we identified some prototype applications developed and suggested for using Blockchain in other environments, such as IoT, smart contracts, smart property, digital content distribution, Botnet, and P2P broadcast protocols. This shows that Blockchain technology is not limited to applications in cryptocurrencies. Instead, the idea of a public ledger and a decentralized environment can be applied to various other applications in different industries, which makes the whole Blockchain research more interesting.

However, we also found a set of different applications developed for the Bitcoin environment, rather than using Blockchain technology in some other environment. Some of the applications were developed for Bitcoin analysis. Applications like BitConeView [51] and BitIodine [23] help users to analyze the Bitcoin network and study how Bitcoin transactions are completed, with a visual presentation. These types of applications can help to understand the essence of Blockchain, and how a decentralized transaction environment actually works. Analysis applications can also help to identify frauds and possible security issues by following the flows of transactions.

Another major direction for applications is security. We found applications where the focus was on Bitcoin mixers. Bitcoin mixing applications, such as CoinParty [47] and CoinShuffle [37] can help the Bitcoin network to become more secure, by adding an extra layer of privacy for the users. These types of applications and solutions will likely increase in the future, considering that security and privacy are the main attributes in a decentralized transaction environment.

### RQ3: What are the current research gaps in Blockchain research?

We were able to identify a few major research gaps. The first gap is that the research on topics such as latency, throughput, size and bandwidth, versioning, hard forks, and multiple forks does not exist in the current literature. This is a major research gap, which requires more research in the future. These topics are not possibly the most interesting topics for researchers at the moment, because the sizes of the current Blockchain applications are relatively small. Bitcoin is currently the largest solution with Blockchain. The number of transactions in Bitcoin is considerably smaller than e.g. in VISA. However, in the future, if Blockchain solutions are used by tens of millions of people and the number of transactions is multiplied drastically, more research on e.g. latency, size and bandwidth, and wasted resources needs to be conducted to ensure scalability.

The second research gap is the lack of research on usability. We identified only papers that discussed usability from the user perspective, not from the developer perspective, as suggested by Swan [1]. For instance, the difficulty of using Bitcoin API has not been tackled yet. This needs to be studied and improved in the future. This could spark more applications and solutions to the Bitcoin environment.

The third research gap is that the majority of current research is conducted in the Bitcoin environment, rather than in other Blockchain environments. Research on e.g. smart contracts needs to be carried out to increase knowledge outside cryptocurrencies. Even though Blockchain was first introduced in the cryptocurrency environment, the same idea can be used in various other environments. Therefore, it is necessary to conduct research on the possibilities of using Blockchain in other environments, because it can reveal and produce better models and possibilities for doing transactions in different industries.

The fourth research gap can be found in the low number of high quality publications in journal level publication channels. Currently most of the research is published in conferences, symposiums and workshops. There is a need for high quality journals where the focus is on Blockchain.

### RQ4: What are the future research directions for Blockchain?

The future research directions for Blockchain are not clear, and it is interesting to see where it is heading. On the other hand, Bitcoin has received a lot of attention as a cryptocurrency, and more people are trading and buying Bitcoins every day. Therefore, it is highly possible that Bitcoin is important as one of the future research topics, and it will attract industry and academia to conduct more research from both business and technical perspectives.

Bitcoin is only one solution using Blockchain technology. There are also a lot of other cryptocurrencies at the moment, competing with Bitcoin to be the world’s primary cryptocurrency. We believe that future research will also include research conducted on other cryptocurrencies. However, at the moment it seems that Bitcoin has by far the largest market share, and it will be a challenge for other cryptocurrencies to compete with it.

However, we believe that future research will not only focus on Bitcoin and other cryptocurrencies, but on other possible applications using Blockchain as a solution. We already found some papers that studied the possibility of using smart contracts, licensing, IoT, and smart properties in the Blockchain environment. We believe that this type of research will have a lot of impact in the future, and can possibly be even more interesting than cryptocurrencies. To use a decentralized environment in e.g. sharing a virtual property could be a solution that revolutionizes the way companies can sell their products. Taking this in consideration, we strongly believe that when Blockchain technology gets adopted more by both industry and academia, it will generate a significant amount of new research.

When more Blockchain solutions are taken in use with larger numbers of users, it will also have an impact on the research done on technical limitations and challenges. In the future, increased sizes and user bases in various Blockchains will trigger the need to conduct more research on the challenges and limitations in topics related to scalability. In addition, the security and privacy of Blockchain will be always a topic for research, when new ways are invented to disturb and attack Blockchain. Although Blockchain is a rather new technology, there already exist profound studies in each problem domain including security and distributed system literature (for example, multi-level authentication technique [56], energy-efficient resource management for distributed systems [55, 57], and etc.). A closer look and adoption of proven solutions would accelerate overcoming current challenges and limitations of Blockchain technology.

### Limitations of the systematic mapping study

The principal limitations of a systematic mapping study are related to publication bias, selection bias, inaccuracy in data extraction, and misclassification [58].

Publication bias refers to the problem that positive results are more likely to be published than negative ones, since negative results take longer to be published or are cited in other publications to a lesser extent [2][58]. To address this issue, we used several well-known scientific databases in the search protocol to find as many papers as possible. This increased the number of papers we were able to find for this mapping study, which to some extent also increased the possibility to find papers with negative results. However, considering that Blockchain technology is rather a new topic in computer science industry and academia, it is possible that research has been conducted in the industry and published as white papers or internally within companies. Therefore, all research conducted on the technical aspects on Blockchain might not be included in this mapping study. However, by using only scientific databases as a source for finding relevant research, we were able to collect papers that were probably of a higher quality.

Selection bias refers to the distortion of statistical analysis owing to the criteria used to select the publications [58]. We addressed this issue by designing our search protocol carefully. We also conducted a pilot search with different keywords, to ensure that we included as many papers as possible in this mapping study. We defined rigorous inclusion and exclusion criteria, to ensure that all the selected papers were part of our research topic, and answered the research questions. However, there is one major limitation that needs to be addressed. Our search protocol included only the term Blockchain. There is a possibility that not all the research related to Blockchain was found due to our search protocol for paper retrieval. Much of the research related to Blockchain concerns economic, legal, or regulation aspects of Bitcoin and its possibilities as a cryptocurrency. Our goal was to study the technical aspects of Blockchain, rather than trying to understand how Bitcoin as a cryptocurrency can work in the real-world environment. Based on our pilot search, we believe that we were able to retrieve a majority of the relevant papers by using only Blockchain as the search term.

Inaccuracy in data extraction and misclassification refer to the possibility that information is extracted differently by different reviewers [58]. We addressed this issue by using three authors in the paper retrieval process. All three authors went through the abstracts of the selected papers, and gave their opinion on including or excluding the paper. In a situation where the opinions did not match, we had a discussion to address whether that specific paper should be included or excluded. In addition, the classifications of the papers were done in several face-to-face meetings, where the three authors discussed and created classifications and mappings to all the 41 selected primary papers.

## Conclusion

Blockchain technology runs the Bitcoin cryptocurrency. It is a decentralized environment for transactions, where all the transactions are recorded to a public ledger, visible to everyone. The goal of Blockchain is to provide anonymity, security, privacy, and transparency to all its users. However, these attributes set up a lot of technical challenges and limitations that need to be addressed.

To understand where the current research on Blockchain technology positions itself, we decided to map all relevant research by using the systematic mapping study process [2]. The goal of this systematic mapping study was to examine the current status and research topics of Blockchain technology. We excluded the economic, law, business, and regulation perspectives, and included only the technical perspective. We extracted and analyzed 41 primary papers from scientific databases. We provide recommendations on future research directions of Blockchain technology based on the current research status as following:

- Continue to identify more issues and propose solutions to overcome challenges and limitations of Blockchain technology.
  The interest on Blockchain technology has been drastically increased since 2013. The cumulative number of papers is increased from 2 in 2013 to 41 in 2015. Majority of the studies has been focused on addressing the challenges and limitations, but there still exist many issues without proper solutions.
- Conduct more studies on scalability issues of Blockchain.
  Most of the current research on the Blockchain technology is focused on security and privacy issues. To be ready for pervasive use of Blockchain technology, scalability issues such as performance and latency have to be addressed.
- Develop more Blockchain based applications beyond Bitcoin and other cryptocurrency systems.
  The current research is focused on Bitcoin system. However, the research also shows that Blockchain technology is applicable for other solutions such as smart contracts, property licensing, voting etc.
- Evaluate the effectiveness of the proposed solutions with an objective evaluation criteria.
  Although several solutions to challenges and limitations have been presented, many of them are just brief idea suggestions and lack concrete evaluation on their effectiveness.

## Supporting Information

S1 Table The full list of selected primary papers. (PDF)

S1 Checklist PRISMA Checklist. (DOC)

S1 Diagram PRISMA Flow diagram. (DOC)
