# Supplementary material for: Where Is Current Research on Blockchain Technology?—A Systematic Review
Source: PLoS One. 2016 Oct 3;11(10):e0163477. doi: 10.1371/journal.pone.0163477 (PMC5047482; doi:10.1371/journal.pone.0163477)
Supplement: S1 Table — (PDF) [file pone.0163477.s001.pdf]

S1 Table. The full list of selected primary papers

ID	Authors	Year	Paper and publication type	Topic
ID01	Koshy et al. [35]	2014	Improvement / conference	Privacy
ID03	Möser et al. [49]	2013	Improvement / conference	Privacy
ID04	Zhang & Wen [22]	2015	Application / conference	Others: New cryptocurrencies
ID05	Feld et al. [53]	2014	Improvement / workshop	Privacy
ID06	Anish [15]	2014	Improvement / conference	Wasted resources
ID07	Decker & Wattenhofer [36]	2014	Report / symposium	Security
ID08	Battista et al. [51]	2015	Improvement / symposium	Usability
ID09	Spagnuolo et al. [23]	2014	Improvement / conference	Usability
ID11	Valenta & Rowan [24]	2015	Improvement / workshop	Privacy
ID12	Bamert et al. [18]	2014	Improvement / workshop	Security
ID13	Ateniese et al. [19]	2014	Improvement / conference	Security
ID14	Vandervort [25]	2014	Improvement / workshop	Usability
ID15	Ziegeldorf et al. [47]	2015	Improvement / conference	Privacy
ID16	Ruffing et al. [37]	2014	Improvement / symposium	Privacy
ID18	Luu et al. [46]	2015	Improvement / conference	Security
ID20	Wan et al. [43]	2015	Application / conference	Others: Smart contracts
ID21	Bos et al. [26]	2014	Report / conference	Security
ID22	Vasek et al. [27]	2014	Report / workshop	Security
ID23	Wang & Liu [21]	2015	Improvement / conference	Wasted resources
ID24	Wilson & Ateniese [44]	2015	Application / conference	Others: Trustworthiness
ID25	Barkatullah & Hanke [54]	2015	Improvement / journal	Wasted resources
ID26	Androulaki & Karame [41]	2014	Improvement / conference	Privacy
ID27	Saxena et al. [28]	2014	Improvement / workshop	Privacy
ID28	Decker & Wattenhofer [52]	2013	Improvement / conference	Security
ID29	Vandervort et al. [29]	2015	Application / workshop	Others: New cryptocurrencies
ID30	Eyal & Sirer [30]	2014	Improvement / conference	Security
ID31	Decker et al. [38]	2015	Improvement / symposium	Usability
ID32	Andrychowicz et al. [31]	2015	Improvement / workshop	Security
ID33	Andrychowicz & Dziembowski [40]	2015	Application / conference	Others: P2P broadcast protocol
ID34	Meiklejohn & Orlandi [32]	2015	Improvement / workshop	Privacy
ID36	Herrera-Joancomart [6]	2015	Report / workshop	Privacy
ID37	Armknecht et al. [42]	2015	Report / conference	Security
ID38	Lim et al. [48]	2014	Report / conference	Security
ID39	Garay et al. [39]	2015	Improvement / conference	Security

ID40	Kishigami et al. [50]	2015	Application / conference	Others: Smart contracts
ID42	Vasek & Moore [33]	2015	Report / conference	Security
ID43	Paul et al. [16]	2014	Improvement / conference	Wasted resources
ID44	Beikverdi & Song [20]	2015	Report / conference	Security
ID45	Mann & Loebenberger [17]	2015	Improvement / workshop	Security
ID46	Bigi et al. [45]	2015	Application / book chapter	Others: Smart contracts
ID47	Ali et al. [34]	2015	Application / workshop	Others: Botnet
