# Supplementary material for: Where Is Current Research on Blockchain Technology?—A Systematic Review
Source: PLoS One. 2016 Oct 3;11(10):e0163477. doi: 10.1371/journal.pone.0163477 (PMC5047482; doi:10.1371/journal.pone.0163477)
Supplement: S1 Diagram — (DOC) [file pone.0163477.s003.doc]

# **S1 Diagram**

# **PRISMA Flow diagram**

**Screening**

**Included**

**Eligibility**

**Identification**

Records identified through database searching
(n = 121)

Additional records identified through other sources
(n = 0)

Records after duplicates removed
(n = 114)

Records screened
(n = 114)

Records excluded
(n = 66)

Full-text articles assessed for eligibility
(n = 48)

Full-text articles excluded, with reasons
(n = 7)

Studies included in qualitative synthesis
(n = 41)

Studies included in quantitative synthesis (meta-analysis)
(n = 41)
